# Metal-Ligand Recognition Index Determination by NMR Proton Relaxation Study

**DOI:** 10.3390/molecules24061050

**Published:** 2019-03-17

**Authors:** Claudia Bonechi, Alessandro Donati, Gabriella Tamasi, Alessio Pardini, Vanessa Volpi, Gemma Leone, Marco Consumi, Agnese Magnani, Claudio Rossi

**Affiliations:** 1 Department of Biotechnology, Chemistry and Pharmacy, University of Siena, Via Aldo Moro 2, 53100 Siena, Italy; claudia.bonechi@unisi.it (C.B.); gabriella.tamasi@unisi.it (G.T.); pardini4@student.unisi.it (A.P.); vanessa.volpi@unisi.it (V.V.); gemma.leone@unisi.it (G.L.); marco.consumi@unisi.it (M.C.); agnese.magnani@unisi.it (A.M.); 2 Centre for Colloid and Surface Science (CSGI), University of Florence, Via della Lastruccia 3, 50019 Sesto Fiorentino, Firenze, Italy; 3 National Interuniversity Consortium of Materials Science and Technology (INSTM), Via G. Giusti 9, 50121 Firenze, Italy; 4 Operative Unit, University of Siena, Campo Verde, Calabria, 53100 Siena, Italy

**Keywords:** NMR spectroscopy, ^1^H-NMR, metal ion recognition index, paramagnetic systems, piroxicam, copper(II)

## Abstract

In this study, we developed and validated a new proposed parameter quantifying the interaction strength between natural and/or synthetic molecules with paramagnetic metal ions. The Metal ion Recognition Index, *Miri*, is a quantitative parameter to describe the proton environment and to define their involvement in the inner and/or outer sphere of the paramagnetic metal ion. The method is based on the analysis of NMR proton spin-lattice relaxation rates of a specific ligand in both the diamagnetic and paramagnetic conditions. The proposed procedure is also useful to calculate the ligand proton spin-lattice relaxation rate in the paramagnetic bound conditions, which is typically very difficult to determine experimentally. *Miri* was used to compare the ligand proton involvement toward different paramagnetic species, in particular the Copper(II)-Piroxicam system. Copper(II)-Piroxicam complex is one of the most active anti-inflammatory and anti-arthritic species. *Miri* provides an opportunity to improve our knowledge of metal-ligand complexes that play a fundamental role in bioinorganic interactions.

## 1. Introduction

Theoretical and experimental studies on metal-ligand interactions are an important field of research [1,2,3,4,5]. Metal-ligand complexes are very important for their applications in medicine, biology, chemistry, agriculture, etc. [6,7,8,9,10,11,12,13]. The formation of complexes between bioactive substances and metal ions, can produce different outcomes: (i) increase the pharmacological effects [14,15], (ii) reduce possible toxic side effects [16,17], (iii) modulate biological activities of both ligands and metal ions [18,19], (iv) contribute to the delivery of the bioactive substances towards their biological targets [20,21]. In the latter case, the metal complexing represents one of the possible methods to couple synergic pharmacological contributions and more efficient delivery systems (e.g., the formation of molecular clusters [22], the encapsulation of the metal complex into lipid or liposomal formulations [23,24,25], and the inclusion of complexes in carrier based hydrogels [26].

Natural and synthetic anti-inflammatory molecules have been widely investigated to find more effective compounds being able to interact with specific receptor sites of the biochemical pathway involved in the activation and maintenance of the inflammatory processes [27,28,29,30]. Metal complexes of the most active anti-inflammatory molecules are good candidates to become potent inhibitors of inflammation processes. The presence of a paramagnetic center in solution was used in the past [31,32,33,34,35,36,37], and more recently to investigate the structural and dynamical properties of metal-biopolymer complexes of peptides and proteins [38,39,40,41,42,43,44,45,46,47].

In the present study, a new method to determine the strength of the interaction between a ligand and a paramagnetic metal ion in solution, was developed and validated. The method is based on the analysis of the paramagnetic contributions to the ligand NMR proton spin-lattice relaxation rates. The experimental results obtained in both diamagnetic and paramagnetic systems were used with developing a new parameter, the “Metal Ion Recognition Index”, *Miri*. This new parameter provides a number of important uses: (a) as a quantitative parameter associated to a specific mathematical and chemical meaning, (b) to discriminate the proton environment around the paramagnetic metal ion and (c) to compare the paramagnetic contributions for different metal-ligand systems. *Miri* can be determined for any stoichiometry of the metal-ligand complex, it is formally related to the thermodynamic equilibrium constant (*K_eq_*). The method offers the possibility to calculate a parameter that is very rarely reported, i.e., the proton spin-lattice relaxation rates in the pure paramagnetic environment (*R*_1*m*_).

The method was validated using Copper(II)-Piroxicam, Cu(II)-Pix, being one of the most active anti-inflammatory and anti-arthritic species [48,49,50,51]. The crystal structure of the Cu(II)-Piroxicam complex is known [49].

## 2. Results and Discussion

### 2.1. Theory

Both the proton relaxation rate and line shape analysis suggested that, in a diamagnetic system, Piroxicam underwent fast motion conditions, i.e., *ω*_0_*τ*_c_ << 1, where *ω*_0_ is the proton Larmor frequency and *τ*_c_ is the correlation time modulating the re-orientational motions. In the presence of the paramagnetic Cu(II) ions, the ligand may exist in either the bound (m) or in the free (f) environments. In the presence of fast chemical exchange between the bound and the free environments, a paramagnetic contribution to the ligand proton relaxation rates can be detected as:

(1) $$R_{1p}=R_{1\exp}-R_{1f}$$

where *R*_1*p*_ is the paramagnetic contribution to the relaxation rate, *R*_1exp_ the experimental relaxation rate in the paramagnetic system, and R_1*f*_ the relaxation rate in the diamagnetic system.

Considering the following equilibrium:

Cu(II)+L→Cu(II)−L

if fast chemical exchange conditions apply, *R*_1exp_ is defined as:

(2) $$R_{1\exp}=\chi_{m}R_{1m}+\chi_{f}R_{1f}$$

where, *R*_1*m*_ is the proton relaxation rate of the paramagnetic complex, and *χ_m_* and *χ_f_* are the molar fractions of the metal complex and the free ligand, respectively. Assuming that *χ_f_* is close to 1 (as the Ligand concentration is usually much higher than the paramagnetic ion concentration):

(3) $$R_{1\exp}=\chi_{m}R_{1m}+R_{1f}$$

and

(4) $$R_{1\exp}-R_{1f}=\chi_{m}R_{1m}$$

or

(5) $$R_{1p}=\chi_{m}R_{1m}$$

*χ_m_* can be defined as:

(6) $$\chi_{m}=\frac{\left[Cu(II)-L\right]}{\left[Cu(II)]+[L\right]}$$

where, [Cu(II)–L], is the concentration of the complex, [L] is the concentration of the free ligand, and [Cu(II)] is the concentration of the free metal ion. Considering, as previously pointed out, that the ligand concentration is much higher than the metal ion concentration, [L] >> [Cu(II)], then:

(7) $$\chi_{m}≅\frac{\left[Cu(II)-L\right]}{\left[L\right]}$$

The thermodynamic equilibrium constant for the Cu(II)-L interaction (at equilibrium) can be defined as:

(8) $$K_{eq}=\frac{\left[Cu(II)-L\right]}{\left[Cu(II)]+[L\right]}=\frac{\left[Cu(II)-L\right]}{\left([Cu{\left(II\right)}_{0}]-[Cu(II)-L])[L\right]}$$

being the [Cu(II)] concentration equal to: [Cu(II)_0_] − [Cu(II)-L]. Then:

(9) $$[Cu(II)-L]=\frac{K_{eq}[Cu{\left(II\right)}_{0}][L]}{1+K_{eq}[L]}$$

Introducing this equation in Equation (7), then:

(10) $$\chi_{m}=\frac{K_{eq}[Cu{\left(II\right)}_{0}][L]}{\left(1+K_{eq}[L])[L\right]}$$

or

(11) $$\chi_{m}=\frac{K_{eq}[Cu{\left(II\right)}_{0}]}{1+K_{eq}[L]}$$

Substituting *χ_m_*, in Equation (5), then:

(12) $$R_{1p}=\frac{K_{eq}[Cu{\left(II\right)}_{0}]}{1+K_{eq}[L]}R_{1m}$$

Assuming the term:

(13) $$\frac{K_{eq}}{1+K_{eq}[L]}R_{1m}=Miri$$

*Miri* is then defined as “Metal Ion Recognition Index”. *Miri* is a constant at constant temperature and constant ligand concentration. From Equation (12):

(14) $$R_{1p}=Miri[Cu{\left(II\right)}_{0}]$$

Equation (14) is the equation of a straight line passing through the origin. Plotting the calculated *R*_1*p*_ values, as a function of [Cu(II)_0_], the value of *Miri* can be calculated from the slope of the linear regression line. *Miri* measures the strength of the paramagnetic interaction, between the metal ion and a specific proton of the ligand molecule. It also helps define the proton environment around the paramagnetic ion.

In case of the presence of more ligand molecules in the metal coordination site, the equilibrium is defined as:

$$nL+Cu(II)\to [Cu(II)-{\left(L\right)}_{n}]$$

Following a similar logic:

(15) $$R_{1p}=\frac{K_{eq}{\left[L\right]}^{n-1}[Cu{\left(II\right)}_{0}]}{1+K_{eq}{\left[L\right]}^{n}}R_{1m}$$

which is an equation of a straight line passing through the origin, when *R*_1*p*_ vs. [Cu(II)_0_] is reported (maintaining both the temperature and ligand concentration [L], constants).

Similarly to Equation (14), Equation (15) can be reduced to:

(16) $$R_{1p}=Miri[Cu{\left(II\right)}_{0}]$$

where *Miri* in this case is:

(17) $$Miri=\frac{K_{eq}{\left[L\right]}^{n-1}}{1+K_{eq}{\left[L\right]}^{n}}R_{1m}$$

Equations (12) and (15) are powerful, in fact Equation (12) can be transformed to:

(18) $$\frac{1}{R_{1p}}=\frac{1+K_{eq}[L]}{[Cu{\left(II\right)}_{0}]K_{eq}R_{1m}}$$

or

(19) $$\frac{1}{R_{1p}}=\frac{1}{[Cu{\left(II\right)}_{0}]K_{eq}R_{1m}}+\frac{\left[L\right]}{[Cu{\left(II\right)}_{0}]R_{1m}}$$

In this case, there is a linear dependence between 1/*R*_1*p*_ and [L], as the paramagnetic ion concentration [Cu(II)_0_], is maintained constant. The slope of the straight line between them leads to the determination of *R*_1*m*_, while the intercept allows for the estimation of the thermodynamic equilibrium constant. In the case of the formation of the metal-ligand complex with a higher stoichiometry, like Cu(II)–(L)*_n_*, Equation (15), can be transformed as:

(20) $$\frac{1}{R_{1p}}=\frac{1+K_{eq}{\left[L\right]}^{n}}{K_{eq}R_{1m}{\left[L\right]}^{n-1}[Cu{\left(II\right)}_{0}]}=\frac{1}{K_{eq}R_{1m}{\left[L\right]}^{n-1}[Cu{\left(II\right)}_{0}]}+\frac{K_{eq}{\left[L\right]}^{n}}{K_{eq}R_{1m}{\left[L\right]}^{n-1}[Cu{\left(II\right)}_{0}]}$$

The linearity of 1/*R*_1*p*_ versus [L] is lost and both *R*_1*m*_ and *K_eq_* cannot be calculated directly from geometrical analysis.

### 2.2. The case of Cu(II)-Piroxicam Complex

**Figure 1:**
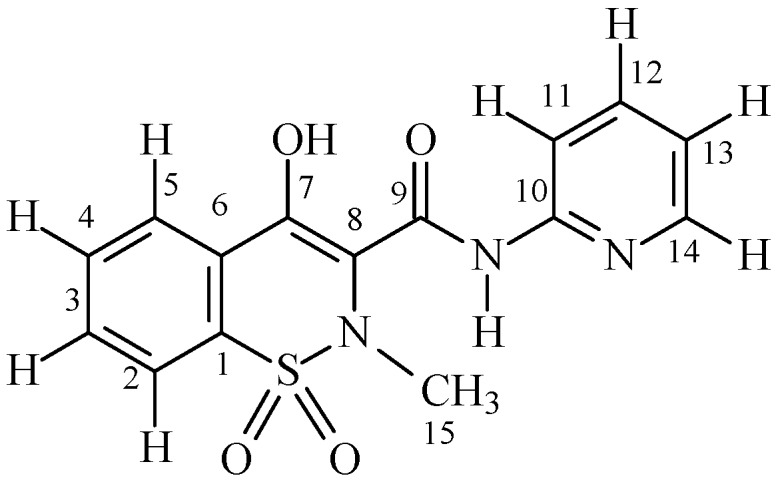
Structure and atom numbering of Piroxicam.

The proton spin-lattice relaxation rates of Piroxicam (Figure 1) in both the diamagnetic and paramagnetic systems are reported in Table 1. The paramagnetic system refers to the proton spin-lattice relaxation rate measured as a function of the copper(II) concentration, in the range of 2 × 10^−5^ to 7 × 10^−4^ mol L^−1^. The paramagnetic contribution to proton spin-lattice relaxation, (*R*_1*p*_ = *R*_1exp_ − *R*_1*f*_; Equation (1)), of Piroxicam proton nuclei are reported in Table 2.

**Figure 2:**
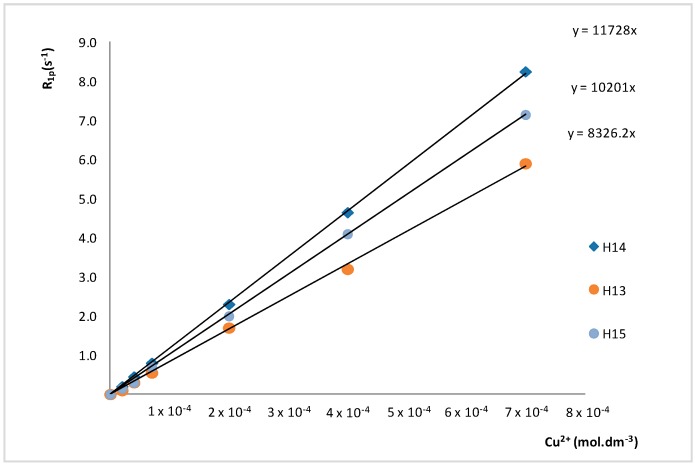
Paramagnetic contributions (*R*_1*p*_) to the proton relaxation rates for the H13, H14, and H15 of 0.1 mol L^−1^ Piroxicam solution versus Cu(II) molar concentration.

The “Metal ion Recognition Index” *Miri*, for the different molecular moieties of Piroxicam was calculated from the linear regression analysis (Figure 2), which resulted in *Miri* values by 11,728, 8326, and 10,201 s^−1^ mol^−1^ L for the H14, H13 and the methyl H15 protons, respectively. These results confirm the validity of Equations (14) and (16) and allow for the quantification of the specific strength of the Cu(II)-Piroxicam complex.

**Figure 3:**
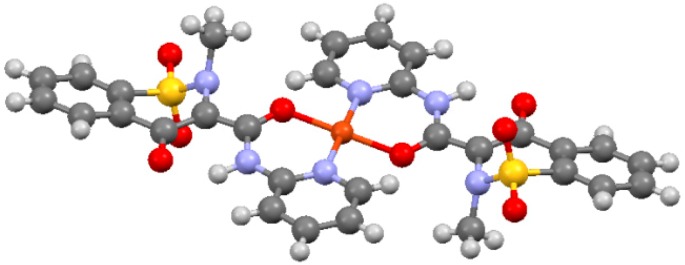
X-ray structure of [Cu^II^(Pix)_2_] complex [49].

We also explored the applicability of equations (19) and (20) to the Cu(II)-Piroxicam system. On the basis of previous studies on the Cu(II)-Piroxicam complex in solution [50,52,53] and on Cu(II)-Piroxicam crystal structure (Figure 3, [49]), we assumed the prevalence of the Cu(II)(Pix)_2_ complex in solution.

As the Piroxicam concentration is a much higher than that of the Copper ion, we consider a predominance of the Cu(II)(Pix)_2_ complex in the present experimental conditions. On the basis of these considerations, Equation (19) cannot be applied and Equation (20), is then modified to:

(21) $$\frac{1}{R_{1p}}=\frac{1}{K_{eq}R_{1m}[Pix][Cu{\left(II\right)}_{0}]}+\frac{\left[Pix\right]}{R_{1m}[Cu{\left(II\right)}_{0}]}$$

Equation (21) presents two terms, the first term can be neglected if the equilibrium constant of the complex, *K_eq_*, is higher than 1 × 10^2^. However, a higher value of *K_eq_* for the complex was previously reported [52,53].

If we neglect the first term, Equation (21) is a linear equation where 1/*R*_1*p*_ varies with Piroxicam concentration. Table 3 reports the paramagnetic contributions to proton spin-lattice relaxation, (*R*_1*p*_ = *R*_1exp_ − *R*_1*f*_; Equation (1)) of Piroxicam proton nuclei as a function of Piroxicam concentration (in the range of 0.25 to 5 × 10^−2^ mol L^−1^), in the presence of Cu(II), 1 × 10^−4^ mol L^−1^ concentration.

**Figure 4:**
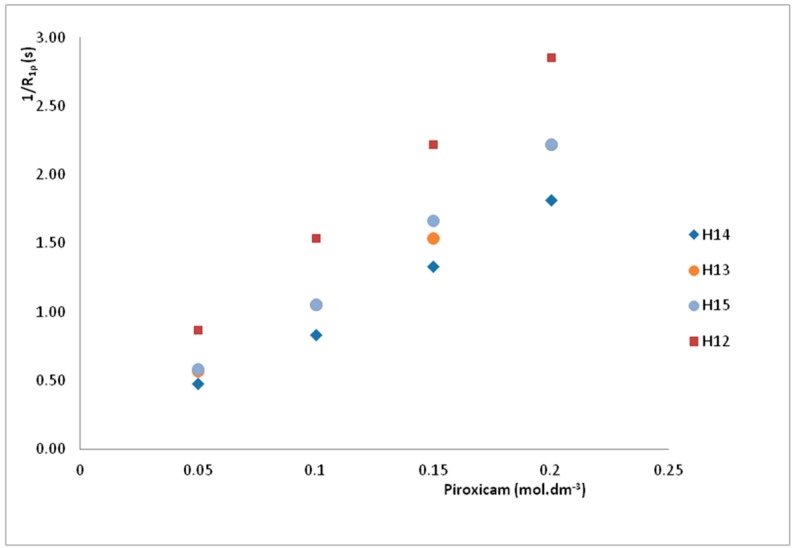
1/*R*_1*p*_ to the proton relaxation rates for the H13, H14, H12, and H15 versus the Piroxicam concentration.

These data can be used to determine *R*_1*m*_, the proton spin-lattice relaxation rate in the pure paramagnetic site. As expected from Equation (21), it is a linear dependence of 1/*R*_1*p*_ on Piroxicam concentration (Figure 4).

The values of *R*_1*m*_, calculated from the slopes of the fitted lines for the H12, H13, H14, and H15 (Table 4) indicated that each proton experience a specific paramagnetic environment as a consequence of the different metal ion proton distances [54].

The calculated spin-lattice relaxation rates in the pure paramagnetic site (*R*_1*m*_), were then used to study the dynamical properties of the metal-ligand complex. The relaxation rate at the paramagnetic site was defined as [55,56,57]:

(22) $$\frac{1}{T_{1,M}}=\frac{2}{15}\frac{\gamma_{I}^{2}g^{2}S(S+1)\beta^{2}}{r^{6}}\left(\frac{3\tau_{c}}{1+\omega_{I}^{2}\tau_{c}^{2}}+\frac{7\tau_{c}}{1+\omega_{S}^{2}\tau_{c}^{2}}\right)+\frac{2}{3}S(S+1){\left(\frac{A}{\hbar }\right)}^{2}\left(\frac{\tau_{e}}{1+\omega_{S}^{2}\tau_{e}^{2}}\right)$$

where the dipolar term originates from the electron-nucleus dipolar contribution, while the scalar one from the modulation of the scalar interaction between the electron spin *S* and the nuclear one *I*. In Equation (22), *ω_I_* and *ω_S_* are the Larmor frequencies of nucleus and electron, respectively (where *ω_I_* >> *ω_S_*), g is the Lande g factor, *β* is the Bohr magneton, *r* is the distance between the nucleus and the paramagnetic species, and (A/ħ) is the electron-nuclear hyperfine coupling constant. Values *τ_c_* and *τ_s_* are correlation times that modulate dipolar and scalar interactions, and are defined as:

(23) $$\tau_{c}^{-1}=\tau_{r}^{-1}+\tau_{s}^{-1}+\tau_{m}^{-1}$$

and

(24) $$\tau_{e}^{-1}=\tau_{s}^{-1}+\tau_{m}^{-1}$$

where *τ_r_* is the rotational correlation time, *τ_s_* the electron spin relaxation time, and *τ_m_* the life time of the nucleus in the bound site.

In the case of paramagnetic systems containing Cu(II) ions in solution, Equation (22) is dominated essentially by the dipolar contribution [58,59,60]. This allows for the determination of the correlation time value τ_c_, in fact from the crystalline structure of the [Cu(II)(Pix)_2_] complex [50], each Cu(II)…proton nuclei distance can be calculated. In this specific case, the distances between the paramagnetic ion Cu(II) and the nuclei H2 and H3 are 4.14, 4.58 Å, respectively. Introducing the metal-ligand distances and the *R*_1*m*_ values in Equation (22), the correlation time (*τ_c_*) modulating the dipolar contribution was calculated. In the present case, this value was determined ranging 3 × 10^−10^–5 × 10^−10^ s. These data are compatible to the value of the rotational correlation time of the complex. This important result confirms that for Cu(II) complexes in solution, the dipolar correlation time is dominated by the molecular tumbling rotation *τ_r_*.

## 3. Materials and Methods

### 3.1. Materials

Piroxicam (4-hydroxy-2-methyl-1,1-dioxo-*N*-pyridinyl-2*H*-1,2-benzothiazine-3-carboxamide) and copper perchlorate hexahydrate Cu(ClO_4_)_2_·6H_2_O, were purchased from Sigma-Aldrich (Milan, Italy) and used without any further purification. The deuterated solvent, DMSO-*d_6_*, was 99.96 atom %D and was also from Sigma-Aldrich.

### 3.2. NMR Measurements

The solutions for the NMR experiments were obtained by dissolving the appropriate amounts of Piroxicam (0.1 mol L^−1^) and Cu(ClO_4_)_2_·6H_2_O in DMSO-d_6_. Paramagnetic purity of the Piroxicam solution was tested by analyzing the NMR proton spin-lattice relaxation rates of both the solvent and water signals.

^1^H-NMR spectra were obtained on a Bruker DRX 600 spectrometer, operating at 600.13 MHz. The proton spin–lattice relaxation rates (R_1_) were measured using the inversion-recovery (180–τ–90–t)*_n_* sequence, where t is the recovery delay after the inversion-recovery perturbation. The R_1_ values were calculated by computer fitting of the relaxation curves. The maximum experimental error in the relaxation rate measurements was ≤5%. All the spectra were processed using the Bruker Software TOPSPIN3.5. The temperature was held constant at 298 ± 1 K for all experiments; and the maximum experimental error on chemical shifts was ≤2%.

## 4. Conclusions

A new approach was developed to study the interaction processes between paramagnetic species and biological and/or synthetic ligands. A new parameter, the metal ion recognition index, *Miri*, was determined by plotting proton spin-lattice relaxation versus the concentration of the paramagnetic ion. The main advantage of this approach, with respect to the measurement of the experimental paramagnetic contribution to nuclear relaxation, is the possibility to define a new parameter, *Miri*. It is formally related to two important chemical parameters, the formation constant of the complex and the relaxation rate of the nuclear species in the pure paramagnetic site, *R*_1*m*_. The developed method provides a new way to their calculation. In the case of 1:1 complexes, both parameters can be calculated. For complexes with more ligands at the metal site, it is only possible to calculate the relaxation rate in the pure paramagnetic site. In the present investigation, combining this information with structural data, obtained from diffraction study of the crystal structure, the rotational correlation time of the complex was also calculated.

The *Miri* is also an easy-to-calculate index giving the opportunity to compare the interaction behavior between a selected metal and different ligands or different metals and a selected ligand.

## Figures and Tables

**Table 1:** Non-selective proton relaxation rate *R*_1*f*_ (s^−1^) of Piroxicam solution (0.1 mol L^−1^) versus Cu(II) concentration. Maximum experimental error, ≤5%.

Proton	δ (ppm)	[Cu] (mol L^−1^)						
		0	2 × 10^−5^	4 × 10^−5^	7 × 10^−5^	2 × 10^−4^	4 × 10^−4^	7 × 10^−4^
H14	8.42	0.40	0.60	0.85	1.20	2.70	5.05	8.65
H2/H5	8.06	0.40	0.45	0.50	0.60	0.80	1.25	1.85
H12	7.99	0.45	0.55	0.65	0.80	1.55	2.65	4.45
H11/H3	7.89	0.50	0.55	0.56	0.70	0.95	1.40	2.27
H4	7.86	0.55	0.60	0.65	0.85	0.95	1.25	1.85
H13	7.28	0.45	0.55	0.75	1.00	2.15	3.65	6.35
H15	2.87	1.10	1.25	1.40	1.80	3.10	5.20	8.25

**Table 2:** Paramagnetic proton relaxation rate contribution *R*_1*p*_ (s^−1^) of Piroxicam solution (0.1 mol L^−1^) versus Cu(II) concentration. Maximum experimental error, ≤5%.

Proton	δ (ppm)	[Cu] (mol L^−1^)					
		2 × 10^−5^	4 × 10^−5^	7 × 10^−5^	2 × 10^−4^	4 × 10^−4^	7 × 10^−4^
H14	8.42	0.20	0.45	0.80	2.30	4.65	8.25
H2/H5	8.06	0.05	0.10	0.20	0.40	0.85	1.45
H12	7.99	0.10	0.20	0.35	1.10	2.20	4.00
H11/H3	7.89	0.05	0.10	0.20	0.45	0.90	1.60
H4	7.86	0.05	0.10	0.30	0.40	0.70	1.30
H13	7.28	0.10	0.30	0.55	1.70	3.20	5.90
H15	2.87	0.15	0.30	0.70	2.00	4.10	7.15

**Table 3:** Paramagnetic proton relaxation rate *R*_1*p*_ (s^−1^) of Piroxicam solutions at several concentrations in the presence of Cu(II) (1 × 10^−4^ mol L^−1^). Maximum experimental error, ≤5%.

Proton	δ (ppm)	[Cu] (mol L^−1^)				
		0.25	0.20	0.15	0.10	5 × 10^−5^
H14	8.42	0.46	0.55	0.75	1.20	2.10
H2/H5	8.06	0.12	0.15	0.20	0.35	0.45
H12	7.99	0.30	0.35	0.45	0.65	1.15
H11/H3	7.89	0.10	0.12	0.17	0.25	0.45
H4	7.86	0.12	0.15	0.20	0.30	0.60
H13	7.28	0.37	0.45	0.65	0.95	1.75
H15	2.87	0.38	0.45	0.60	0.95	1.70

**Table 4:** Calculated *R*_1*m*_ values from linear regression analysis of the data reported in Figure 2, for H14, H12, H13, and H15 protons of Piroxicam.

Proton	δ (ppm)	*R*_1*m*_ (s^−1^)
H14	8.42	1129 ± 55
H12	7.99	730 ± 36
H13	7.28	943 ± 46
H15	2.87	917 ± 44
